# Lung health and exposure to air pollution in Malawian children (CAPS): a cross-sectional study

**DOI:** 10.1136/thoraxjnl-2018-212945

**Published:** 2019-08-29

**Authors:** Sarah Rylance, Rebecca Nightingale, Andrew Naunje, Frank Mbalume, Chris Jewell, John R Balmes, Jonathan Grigg, Kevin Mortimer

**Affiliations:** 1 Department of Clinical Sciences, Liverpool School of Tropical Medicine, Liverpool, UK; 2 Lung Health Group, Malawi-Liverpool-Wellcome Trust Clinical Research Programme, Blantyre, Malawi; 3 CHICAS, University of Lancaster, Lancaster, UK; 4 Environmental Health Sciences Division, University of California Berkeley, Berkeley, California, USA; 5 Centre for Child Health, Queen Mary University London, London, UK; 6 Aintree University Hospitals NHS Foundation Trust, Liverpool, UK

**Keywords:** paediatric lung disaese, asthma epidemiology, paediatric asthma, lung physiology

## Abstract

**Background:**

Non-communicable lung disease and exposure to air pollution are major problems in sub-Saharan Africa. A high burden of chronic respiratory symptoms, spirometric abnormalities and air pollution exposures has been found in Malawian adults; whether the same would be true in children is unknown.

**Methods:**

This cross-sectional study of children aged 6–8 years, in rural Malawi, included households from communities participating in the Cooking and Pneumonia Study (CAPS), a trial of cleaner-burning biomass-fuelled cookstoves. We assessed; chronic respiratory symptoms, anthropometry, spirometric abnormalities (using Global Lung Initiative equations) and personal carbon monoxide (CO) exposure. Prevalence estimates were calculated, and multivariable analyses were done.

**Results:**

We recruited 804 children (mean age 7.1 years, 51.9% female), including 476 (260 intervention; 216 control) from CAPS households. Chronic respiratory symptoms (mainly cough (8.0%) and wheeze (7.1%)) were reported by 16.6% of children. Average height-for-age and weight-for-age z-scores were −1.04 and −1.10, respectively. Spirometric abnormalities (7.1% low forced vital capacity (FVC); 6.3% obstruction) were seen in 13.0% of children. Maximum CO exposure and carboxyhaemoglobin levels (COHb) exceeded WHO guidelines in 50.1% and 68.5% of children, respectively. Children from CAPS intervention households had lower COHb (median 3.50% vs 4.85%, p=0.006) and higher FVC z-scores (−0.22 vs −0.44, p=0.05) than controls.

**Conclusion:**

The substantial burden of chronic respiratory symptoms, abnormal spirometry and air pollution exposures in children in rural Malawi is concerning; effective prevention and control strategies are needed. Our finding of potential benefit in CAPS intervention households calls for further research into clean-air interventions to maximise healthy lung development in children.

**Key messages**

**What is the key question?**

- Is the high burden of chronic respiratory morbidity and household air pollution exposure described in Malawian adults, also seen in children, and would a cleaner-burning biomass-fuelled cookstove intervention have a positive effect on lung function in early life?

**What is the bottom line?**

- We found a substantial burden of chronic respiratory symptoms, spirometric abnormalities and carbon monoxide exposures among young children living in rural Malawi, together with a signal of beneficial effect of a cookstove intervention on carboxyhaemoglobin and forced vital capacity.

**Why read on?**

- Chronic respiratory morbidity in adulthood is influenced by lung health in early life—greater understanding of contributing factors is vital to promote healthy lung development during childhood.

## Introduction

Non-communicable lung diseases are major global health priorities across the life course.^1 2^ Asthma is the the most common chronic disease of childhood and one of the the most common chronic diseases of adulthood, affecting around 358 million people while COPD affects 174 million people, worldwide.^3^

Although most of the children and adults with these conditions live and die in low-income countries and middle-income countries (LMICs), the majority of the research into these conditions is done in high-income countries. Research is especially scarce in the LMICs of sub-Saharan Africa where limited studies suggest the prevalence of childhood asthma is increasing in urban settings, and that children with symptoms of asthma are likely to be severely symptomatic.^4 5^ In adult populations, Burden of Obstructive Lung Diseases (BOLD) studies from countries in sub-Saharan Africa, including sites in urban and rural Malawi, have found a high burden of impaired lung function—particularly low forced vital capacity (FVC)^6–8^—which is concerning given the association between low FVC and mortality in other populations.^9^

In these same sub-Saharan African populations, there is widespread reliance (by around 700 million people) on inefficiently burned solid fuels for cooking, heating and lighting.^10^ Studies in rural Malawi report exclusive biomass fuel use (wood, crop waste and charcoal) with households using traditional ‘open-fire’ cooking methods.^11^ The widespread exposure of children to pollutants such carbon monoxide (CO) and particulate matter, resulting from incomplete fuel combustion, is particularly concerning. Household air pollution has been suggested as a potential contributing factor in the development of non-communicable lung diseases in low-income countries.^12^ However, the links between household air pollution exposure, new-onset asthma in children and obstructive lung disease in adults, are unclear, with controversy over the interpretation of available data.^13–17^ Environmental exposures, including inhaled pollutants, during periods of lung growth and development may lead to irreversible long term deficits in adult lung function.^18 19^

In this context, the Cooking and Pneumonia Study (CAPS) was done to determine whether an intervention comprising two cleaner burning biomass-fuelled cookstoves and a solar charger would reduce the incidence of Integrated Management of Childhood Illness-defined pneumonia in children under the age of 5 years in rural Malawi compared with continuation of traditional cooking methods.^11^ CAPS recruited households from village clusters in Chikhwawa between December 2013 and February 2016. The primary intention-to-treat analysis found no difference in pneumonia incidence between the two trial arms. Recently reported secondary analyses in adults from a subset of CAPS households found no difference in chronic respiratory symptoms, lung function or personal air pollution exposures between participants from the intervention and control groups.^20^ That said, median exposure to fine particulate matter (PM_2.5_) was 71 µg/m^3^, well above WHO annual and 24 hours guidelines.

Is it not known whether the same pattern of respiratory symptoms, spirometric abnormalities and air pollution exposures would be seen in children as in adults or whether the CAPS intervention would have beneficial effects on any of these outcomes in children? In this paper we report the findings of a cross-sectional study, conducted in the same village communities as CAPS, which set out to: (1) measure the prevalence and determinants (including measured exposure to household air pollution) of non-communicable lung disease in a population representative sample of children in rural Malawi and (2) conduct an analysis comparing lung function between young children in the intervention group and those in the control group in CAPS. Some of the data have been previously presented in abstract form.^21^

## Methods

### Study design

We conducted a cross-sectional study of the prevalence and determinants of non-communicable respiratory disease among children living in Chikhwawa District, Malawi.

### Setting

Chikhwawa is a rural area, located in the Southern Region of Malawi on the Shire River, 50 km from the nearest city, Blantyre. The population consists largely of subsistence farmers living in village communities and is highly vulnerable to climatic shocks, having experienced flooding, crop failures and famine in recent years. Infectious diseases (malaria, pneumonia and gastroenteritis), HIV/AIDS, malnutrition and limited access to basic healthcare contribute to high childhood mortality rates, although a considerable reduction in the mortality rate for children under 5 years old has been seen in Malawi over the past 25 years.^22^

### Participants

Following widespread community engagement events, children aged between 6 and 8 years, living in households that had taken part in CAPS and BOLD-Chikhwawa were identified by local community advisors and invited to participate if the child’s parent/guardian gave written informed consent (or witnessed thumbprint for those unable to read and write). Exclusion criteria were current treatment for tuberculosis, current acute respiratory infection (defined as cough of <1-week duration, associated with fever and/or increased work of breathing) and other contraindications to spirometry (chest or abdominal pain, haemoptysis). We recruited all children from the study area meeting the eligibility criteria.

### Procedures

Fieldworkers visited the children in the community to administer an electronic questionnaire, and assess anthropometry, lung function, and personal exposure to household air pollution. An electronic questionnaire was administered in Chichewa, the local language, detailing respiratory symptoms and potential contributing factors. Core written questions from the International Study of Asthma and Allergy in Children (ISAAC) were included, which had been forward and back-translated.^23^ Height, weight and mid-upper arm circumference (MUAC) were measured according to standardised protocols. Height and weight were interpreted using the WHO 2007 child growth standards.^24^ MUAC was used to assess nutritional status.^25^

Prebronchodilator and postbronchodilator spirometry was performed by BOLD centre-certified technicians, according to American Thoracic Society/European Respiratory Society (ATS/ERS) standards using an Easy On-PC Spirometer (ndd Medical Technologies; Zurich, Switzerland).^26^ Regular calibration was performed according to the manufacturer’s instructions. The highest forced expiratory volume in one second (FEV_1_) and FVC measurements for each participant were selected (from a maximum eight attempts), before and after administration of 400 µg inhaled salbutamol, via Volumatic spacer. Reversibility was defined as ≥12% improvement between prebronchodilator and postbronchodilator FEV_1_.

Spirometry over-reading was performed by two independent reviewers. Two sets of ATS/ERS standards (aged 4–6 years and aged seven and above) are relevant for the children in this study.^26 27^ As the age range of our study children overlaps both sets of standards, and to maximise the use of spirometric data collected, we defined acceptable (grade C) quality as two traces within 150 mL or 10% (online supplementary table S1).

10.1136/thoraxjnl-2018-212945.supp1 Supplementary data

Carboxyhaemoglobin level (COHb) was measured at a single time-point using a Rad-57 pulse CO-oximeter (Masimo Corporation, California, USA). Performance verification was ensured at study outset, according to the manufacturer service manual. To assess personal CO exposure levels, children wore an EasyLog CO USB data logger (Lascar Electronics, Wiltshire, UK), for up to 48 hours, starting immediately after the field visit.

### Variables

Clinical outcomes were presence or absence of symptoms, as assessed by the following questions; *Chronic cough*: defined by a positive response to both ‘Does your child usually have a cough when they don’t have a cold?’ and ‘Are there months in which they cough on most days?’; *Current wheeze*: ‘Has your child had wheezing or whistling in the chest in the past 12 months?’; *Severe asthma:* current wheeze, and ≥4 attacks of wheeze, or ≥1 night per week sleep disturbance from wheeze, or wheeze affecting speech, in the past 12 months; *Shortness of breath*: a composite outcome, positive if children were reported to be breathless during normal daily activities or on minimal exertion; *Any respiratory symptom*: a composite outcome, positive if a participant was reported to have any of the previously described symptom outcomes.

Continuous FEV_1_ and FVC values were used in the primary analysis. Standardised z-scores and lower limits of normal (LLN) for FEV_1_, FVC and FEV_1_/FVC were derived from the GLI 2012 reference equations for African-Americans, which provide race-specific and sex-specific reference values, taking into account height and age.^28^

Personal CO exposure monitoring data were not analysed if <24 hours were recorded. To allow comparison of varying lengths of recording, all data were truncated at 24 hours for the final analysis.

Potential effect modifiers included were height (cm), weight (kg), age and sex.

### Study size

We calculated a sample of 600 participants (300 male, 300 female) would estimate the prevalence of non-communicable lung disease in each sex stratum with a precision (95% CI) of ±3.3 to ±5.0% (assuming a prevalence of 10%–25%). To allow for unequal sex distributions, refusals and inability to provide spirometry of acceptable quality, we aimed to recruit 1000 children.

### Statistical analysis

Descriptive analysis was performed, using Student’s t-test and Pearson’s χ^2^ to compare continuous and categorical data. For population proportions, Wald-type SEs were calculated, assuming a binomial distribution. Bivariate associations between spirometric and clinical outcomes, and variables including CO, COHb, hospital admission for respiratory illness during infancy, and CAPS allocation were explored. Harmonic regression was used to account for any possible effect of seasonality on the outcome measures. This was implemented by including sinusoidal functions (sine and cosine terms) of time with a period of 1 year. Linear multivariable regression was used to estimate the association between exposures and continuous lung function values (FEV_1_ and FVC). Multivariable logistic regression models were constructed for dichotomous clinical outcomes. All models included age, sex, height and weight *a priori*, and variables with a p value <0.2 on bivariate analysis. A backward stepwise regression technique was used to develop multivariable models. An analysis was conducted to compare FEV_1_, FVC and FEV_1_/FVC, symptom prevalence and exposure variables between the intervention and control groups of CAPS. CO was log_10_ transformed for inclusion in linear models to ensure normality of residuals.

Analyses were conducted using R V.3.4.1 statistical software.^29^

### Role of the funding source

The funders had no role in the study design, data collection, analysis, interpretation or writing of the report. The corresponding author had full access to all the study data and had final responsibility for the decision to submit for publication.

Ethical approval was given by the College of Medicine Research Ethics Committee in Malawi (reference P.07/16/1994) and Liverpool School of Tropical Medicine Research Ethics Committee in the UK (reference 16–040).

## Results

**Figure 1:**
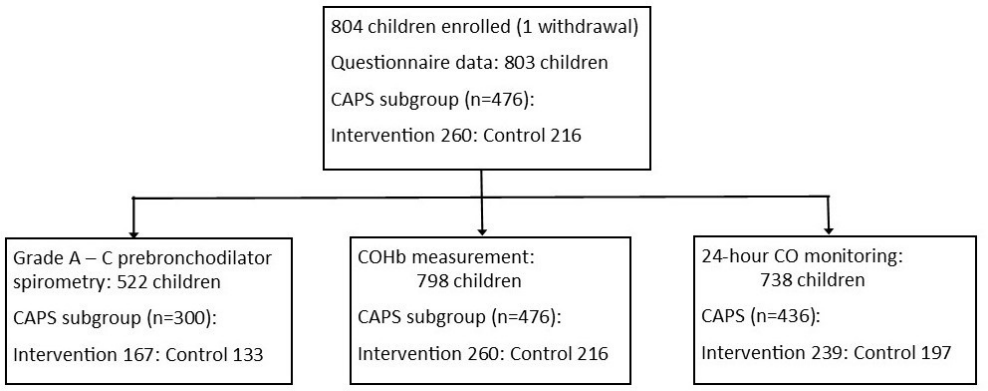
Participant recruitment flow diagram. CAPS, Cooking and Pneumonia Study; CO, carbon monoxide; COHb, carboxyhaemoglobin level.

Between February and December 2017, we approached 886 children of whom 804 were confirmed to be eligible and were recruited (79/82 were outside the eligible age range; 3/82 guardians declined to consent). Questionnaire data were collected for all but one participant who withdrew from the study shortly after giving consent. Anthropometry, spirometry, COHb measurement and personal CO monitoring were done on 99.9% (802/803), 99.9% (802/803), 99.4% (798/803) and 99.3% (797/803) of these participants, respectively. Grade A–C prebronchodilator traces were achieved in 65% (522/802) of the children. The duration of CO monitoring was 24 and 48 hours for 91.9% (738/803) and 79.5% (638/803) children, respectively. There were 476 (260 intervention and 216 control) children from households included in CAPS (figure 1).

The mean age (SD) of participants was 7.13 (0.77) years and 417 (51.9%) participants were female. Most (700 (87.2%)) were attending primary school. The mean (SD) height-for-age and weight-for-age z-scores were −1.04 (0.90) and −1.10 (0.89), respectively. Mean (SD) MUAC was 15.98 (1.26) cm (table 1). No children met the criteria for severe or moderate acute malnutrition, but 11/789 (1.4%) children were ‘at risk for acute malnutrition’.

**Table 1:** Demographics and clinical characteristics (n=803)

Female, n (%)	417 (51.9)
Age, mean (SD) years	7.13 (0.77)
School attendance, n (%)	700 (87.2)
Anthropometry	
Weight-for-age z-score, mean (SD)	−1.10 (0.89)
Height-for-age z-score, mean (SD)	−1.04 (0.90)
MUAC, mean (SD) cm*	15.98 (1.26)
Chronic respiratory symptoms	**n (%)**
Wheeze ever	97 (12.1)
Current wheeze (in the past 12 months)	57 (7.1)
Severe asthma (in the past 12 months)	31 (3.9)
Wheeze with exercise	44 (5.5)
Dry cough at night	145 (18.1)
Chronic cough	64 (8.0)
Chronic sputum production	13 (1.6)
Chronic shortness of breath	49 (6.1)
Any chronic respiratory symptom	133 (16.6)

*MUAC measurement available for 789 participants.

MUAC, mid-upper arm circumference.

Chronic respiratory symptoms were reported by 133 (16.6% (SE 1.3)) children, most commonly cough (8.0% (SE 1.0)), and current wheeze (7.1% (SE 0.9)) (table 1). One-fifth (159/803) of children had been admitted to hospital with respiratory symptoms in the past; on one (9.7%), two (6.1%) and three or more (4.0%) occasions. Admission for a respiratory problem during the first year of life was reported for 70 (8.7%) children. Antibiotic use for a chest problem in the last year was common, reported for 112 (13.9%) children, with 69 (8.6%) receiving these on more than one occasion. Half (54.4%) of children with current wheeze had symptoms of severe asthma, representing 3.9% of children overall. Of these, 22 (71.0%) had a previous hospital admission, and 10 (32.2%) missed school due to breathing problems. Very few (0.4%) children had previously been treated for tuberculosis, and 2.0% (6/307) of children who had been tested for HIV were HIV-positive.

Children producing grade A–C spirometry were older than those with unacceptable traces (mean age 7.23 vs 6.96 years, p<0.001); otherwise there were no significant differences in growth parameters and respiratory symptoms between the two groups (online supplementary table S2). Overall, participants had a mean (SD) FEV_1_ z-score −0.48 (0.93) and mean (SD) FVC z-score of −0.30 (0.96). Children from CAPS intervention households had higher FVC z-scores than those from control households (−0.22 vs −0.44, p=0.05). Prebronchodilator spirometric abnormalities were found in 68/522 (13.0%) of children; 7.1% with low FVC and 6.3% obstruction (table 2). Postbronchodilator spirometry was attempted by 706 children, with 72% (505/706) producing grade A–C traces. Both prebronchodilator and postbronchodilator traces were available for 432 children, 26 of whom had a prebronchodilator FEV_1_/FVC ratio below the LLN which was reversible in 8 (30.7%).

**Table 2:** Prebronchodilator lung function parameters for participants with grade A–C spirometry, including the CAPS subgroup

	Participants with A–C spirometry N=522	CAPS intervention arm N=167	CAPS control arm N=133	Intervention versus control *
FEV_1_ z-score, mean (SD)	−0.48 (0.93)	−0.41 (0.92)	−0.60 (0.97)	P=0.10
FVC z-score, mean (SD)	−0.30 (0.96)	−0.22 (0.97)	−0.44 (0.98)	P=0.05
FEV_1_/FVC z-score, mean (SD)	−0.38 (0.90)	−0.40 (0.91)	−0.34 (0.93)	P=0.57
FVC<LLN, n (%)	37/522 (7.1%)	11/167 (6.6%)	12/133 (9.0%)	P=0.57
Obstructive spirometry FEV_1_/FVC<LLN, n (%)	33/522 (6.3%)	11/167 (6.6%)	10/133 (7.5%)	P=0.93
Abnormal spirometry (low FVC, obstruction, mixed), n (%)	68/522† (13.0%)	21/167‡ (12.6%)	22/133 (16.5%)	P=0.42

*Comparison of means using Student’s t-test; comparison of proportions using Pearson’s χ^2^ test.

†Mixed pattern in two participants.

‡Mixed pattern in one participant.

CAPS, Cooking and Pneumonia Study; FEV_1_, forced expiratory volume in one second; FVC, forced vital capacity.

**Figure 2:**
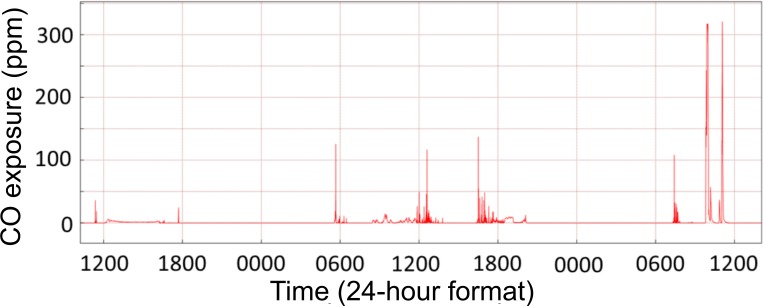
Example of a typical 48 hours CO monitoring trace. CO, carbon monoxide.

**Figure 3:**
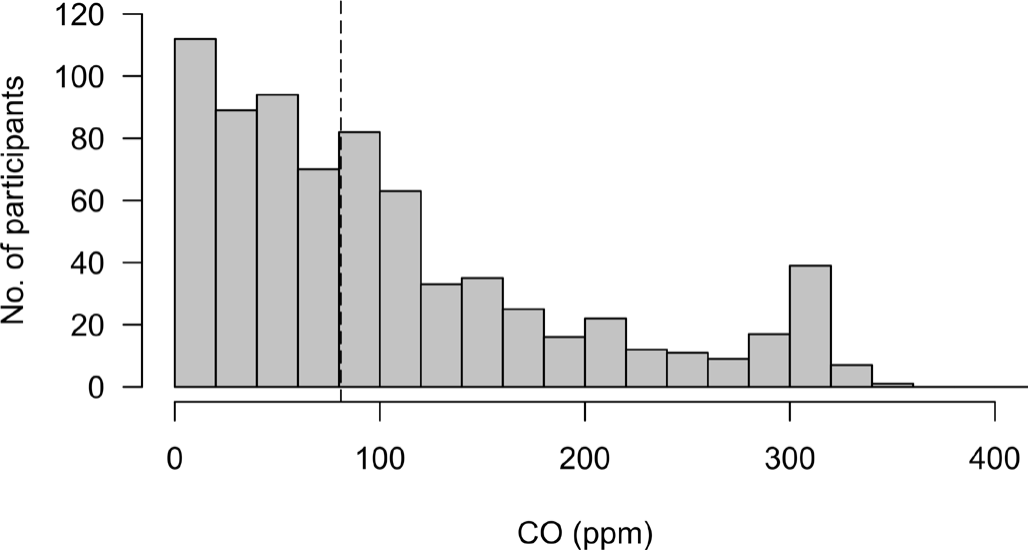
Maximum CO levels recorded during monitoring period for 738 participants. Dashed line represents who recommended indoor exposure guideline for a 15 min time period. CO, carbon monoxide.

**Figure 4:**
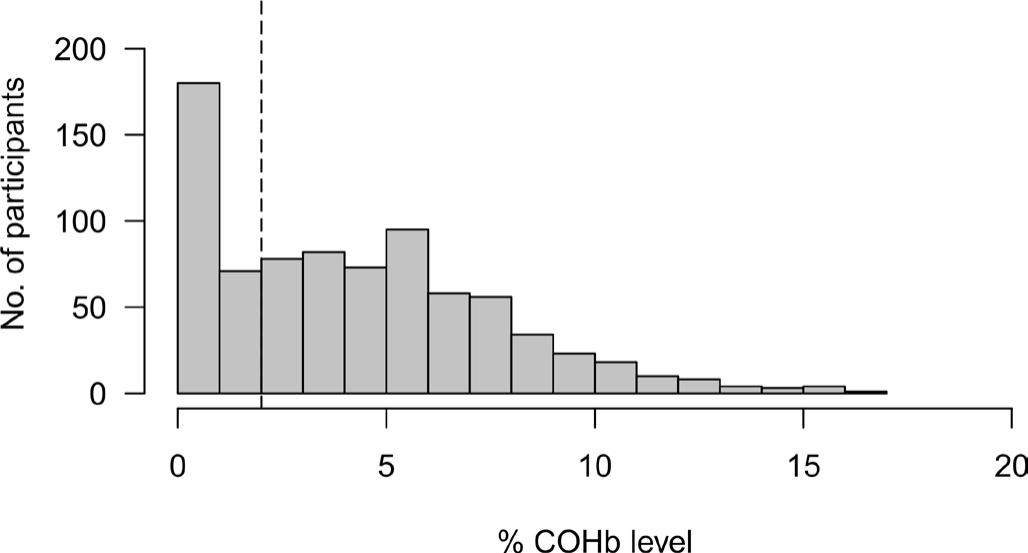
Percentage of COHb level for 798 participants. Dashed line represents the WHO COHb guideline. COHb, carboxyhaemoglobin level.

Personal CO monitoring showed considerable variation in exposure throughout the monitored period (figure 2). Mean exposure levels ranged from 0 to 15.1 parts per million (ppm), with a median CO exposure of 0.20 ppm (IQR 0.07–0.54). Peaks exceeding the 15 min indoor WHO guideline (81 ppm; 100 mg/m^3^) were observed in 370/738 (50.1%) of participants (figure 3).^30^ Median %COHb was 4.00 (IQR 1.50–6.50). 68.5% of participants had a level greater than 2%, and 6.0% greater than 10% (figure 4). We found no association between respiratory symptoms or spirometric indices and personal CO and COHb measurements in bivariate analyses and therefore these variables were not carried forward into multivariable analysis. In logistic multivariable analysis, chronic cough (OR 2.63 (95% CI 1.13 to 6.12)), current wheeze (OR 5.48 (95% CI 2.45 to 12.26)) and symptoms of severe asthma (OR 6.36 (95% CI 2.34 to 17.28)) were all associated with hospital admission during infancy (table 3). We found no association between respiratory symptoms and spirometric indices in bivariate or multivariable analysis (table 3).

**Table 3:** OR (95% CI) for chronic respiratory symptoms estimated by multivariable logistic regression (n=522)

	Cough	Current wheeze	Severe asthma	Shortness of breath
Age (years)	0.72 (0.48 to 1.06)	0.55 (0.31 to 0.96)*	–	–
Sex	–	–	–	–
Height (cm)	–	1.07 (0.99 to 1.17)	1.18 (1.04 to 1.35)*	1.06 (0.98 to 1.14)
Weight (kg)	–	–	0.72 (0.54 to 0.94)*	–
Admission during infancy	2.63 (1.13 to 6.12)*	5.48 (2.45 to 12.26)†	6.36 (2.34 to 17.28)†	–
FEV_1_ (l)‡	–	0.14 (0.01 to 1.72)	0.04 (0.00 to 1.09)	1.24 (0.01 to 1.17)

*Significant at 0.05 level.

†Significant at 0.001 level.

‡Prebronchodilator FEV_1_.

FEV_1_, forced expiratory volume in one second.

In the analysis comparing intervention and control groups, we found statistically significant associations between the intervention arm and both FVC (coefficient estimate 0.04 (95% CI 0.00 to 0.07)), and COHb level (coefficient estimate −0.89 (95% CI −1.53 to 0.26) (table 4A). We found no significant differences between CAPS arms for growth parameters (table 4A) or chronic respiratory symptom rates (table 4B).

**Table 4A:** CAPS secondary trial analyses: mean or median values, with linear model coefficient estimates (95% CI) for continuous outcomes

	Intervention	Control	Intervention versus control	P value
FEV_1_, mean (SD) L*	1.02 (0.18)	0.97 (0.19)	0.02 (−0.01 to 0.06)	0.135
FVC, mean (SD) L*	1.16 (0.21)	1.09 (0.21)	0.04 (0.00 to 0.07)	0.033
FEV_1_/FVC, mean (SD) *	0.88 (0.06)	0.89 (0.06)	−0.01 (−0.02 to 0.01)	0.411
%COHb, median (IQR)†‡	3.50 (1.00 to 6.00)	4.85 (2.00 to 7.00)	−0.89 (−1.53 to −0.26)	0.006
Mean CO ppm, median (IQR)§‡¶**	0.18 (0.05 to 0.55)	0.20 (0.08 to 0.52)	0.03 (−0.35 to 0.42)	0.857
Weight-for-age z-score, mean (SD)†	−1.20 (0.89)	−1.06 (0.85)	−0.13 (−0.29 to 0.02)	0.096
Height-for-age z-score, mean (SD)†	−1.10 (0.84)	−1.06 (0.93)	−0.04 (−0.20 to 0.12)	0.624
MUAC, mean (SD) cm††	15.92 (1.29)	15.94 (1.30)	−0.02 (−0.26 to 0.21)	0.846

*Spirometry data for 300 participants; 167 intervention, 133 control. FEV_1_, FVC and FEV_1_/FVC adjusted for age, sex, height and weight in regression model.

†COHb, height and weight data for 476 participants; 260 intervention, 216 control.

‡Adjusted for seasonality in linear regression model.

§24 hours CO monitoring for 436 participants; 239 intervention, 197 control.

¶Log_10_ CO values used in linear regression model.

**Mean exposure was estimated over the monitoring period per individual, the median of these values (and IQR) is presented for the study population.

††MUAC for 466 participants; 260 intervention, 206 control.

CO, carbon monoxide; COHb, carboxyhaemoglobin level; FEV_1_, forced expiratory volume in one second; FVC, forced vital capacity; MUAC, mid-upper arm circumference.

**Table 4B:** CAPS secondary trial analyses: proportions and OR (95% CI) for symptom outcomes (n=476)

	Intervention n=260	Control n=216	Intervention versus control	P value
Cough, n (%)	30 (7.7%)	18 (8.3%)	0.92 (0.47 to 1.78)	0.797
Current wheeze, n (%)	19 (7.3%)	17 (7.9%)	0.92 (0.47 to 1.82)	0.817
Severe asthma, n (%)	11 (4.2%)	10 (4.6%)	0.91 (0.38 to 2.19)	0.833
Shortness of breath, n (%)	13 (5.0%)	14 (6.5%)	0.75 (0.34 to 1.63)	0.471
Any respiratory symptom, n (%)	37 (14.2%)	40 (18.5%)	0.72 (0.44 to 1.18)	0.193

## Discussion

This is one of the first studies to report lung function and personal household air pollution exposure, measured concurrently in young children, and it was conducted in the context of the largest trial of a cleaner-burning cookstove intervention to date. Among children living in rural Malawi, we found that; one in six reported chronic respiratory symptoms; over half with current wheeze had severe symptoms; anthropometric and lung function parameters were generally decreased compared with global reference ranges; the majority of children had COHb levels above WHO recommended guidelines; and half of children exceeded WHO guidelines for CO exposure (100 mg/m^3^), during 24 hours monitoring.^10^ Overall, we found no evidence of an association between CO exposure and respiratory symptoms or lung function. However, children from CAPS intervention households had higher FVC z-scores and lower COHb levels than controls.

There are limited data regarding chronic respiratory symptoms in children from Africa, and particularly rural settings. One study from rural Senegal reported similar rates with 9% current wheeze and 5% severe asthma among children aged 5–8 years.^31^ Studies from urban settings in sub-Saharan Africa, including ISAAC sites, reported rates of current wheeze in 5%–16% of young children, with symptoms of severe asthma in half of these.^4 32–34^ Globally 11.5% of children aged 6–7 years have current wheeze, and 4.9% have symptoms of severe asthma; severe symptoms are seen in one-third of children with current wheeze in Europe.^4^ The high rates of severe symptoms seen in low-income countries are concerning, and likely reflect multiple challenges within healthcare systems, which are better equipped to manage acute episodes relating to infectious diseases, rather than chronic non-communicable conditions. In keeping with this, recent research from Nigeria and South Africa has reported high rates of under-diagnosed and untreated asthma in schoolchildren.^35 36^

We found decreased lung function parameters in this study, comparable to values reported for community controls in a recent study exploring long-term outcomes after severe acute malnutrition, at the referral hospital in Blantyre, Malawi.^37^ These lung function deficits, when compared with international reference ranges, may reflect host and environmental factors such as undernutrition, frequent respiratory infections, low birth weight, exposure to pollutants in utero and early life, which can have adverse effects on lung growth and development.^38–42^ No children in this study were acutely malnourished (as defined by MUAC measurement), although other anthropometric parameters (weight-for-age and height-for-age z-scores) were reduced compared with international standards, suggesting a level of chronic undernutrition in this community. There are limited data regarding normal lung function in healthy African paediatric populations, and consequently it is difficult to understand the clinical significance of these apparent spirometric deficits.^43^ Further research is needed to describe optimal lung growth in African populations, and determine the morbidity and mortality associated with lung function abnormalities.^44^

Consistent with our previous findings in Chikhwawa, we noted exposure to high peaks of CO, reaching up to three times the WHO guidelines around cooking times, although mean and median levels were low; median CO 1.23 ppm (IQR 0.79–1.93) in adults and mean CO 1.27 ppm (SD 2.79) in younger children.^20 45^ Median CO exposure levels were lower (0.20 ppm (IQR 0.07–0.54) in our older paediatric population perhaps reflecting long periods of time that children spend away from the home environment during the school day. Cookstove trial analyses exploring adult lung function as a secondary outcome have found no evidence of intervention benefit.^20 46 47^ Paediatric lung function outcomes in cookstove trials are inconclusive, but signal a possible beneficial effect of the interventions. Secondary analysis from the RESPIRE trial found decreased lung growth at around 5 years of age (measured by peak expiratory flow), associated with delayed chimney stove installation, although there was no association between lung function at age five and measured personal CO exposure during the first 18 months of life.^48^ The GRAPHS birth cohort in rural Ghana recently reported an association between prenatal CO exposure and infant lung function at 30 days of life, with an increased effect of exposure on female infants.^49^ Cross-sectional studies from Nigeria have described decreased lung volumes (FEV_1_ and FVC) and increased asthma symptoms in children with self-reported exposure to biomass cooking fuels.^36 50^

The association between CAPS intervention group and higher FVC is interesting, given the lack of evidence for an association between lung function and CO exposure or COHb level. This positive finding must be interpreted cautiously as it is the result of exploratory secondary analyses, unadjusted for multiplicity and therefore may be due to chance. However, when taken with the second signal of a potential effect, lower COHb observed in the intervention group, the results may be evidence of a genuine impact. We may have observed a benefit among our participants, who were aged 3–6 years during the CAPS trial period, in contrast to findings from adult populations, because the early childhood years represent a key period for lung development. There is rapid alveolar expansion and resulting lung growth during the first 2 years of life, which stabilises around 8 years of age.^51^ Alveolar number is reflected by FVC in childhood and so it is biologically plausible that we might see improved lung function in children from the intervention arm; the apparent difference of 70 mL in mean FVC between CAPS groups represents approximately 6% of a child’s lung volume. Furthermore, young children have increased susceptibility to air pollutants, exhibiting increased deposition of particles in the lung, due to physiological and anatomical factors.^52^ CO exposure measures do not appear to be associated with lung function or respiratory symptoms—perhaps CO is an inadequate proxy for other pollutants of interest, such as PM_2.5_ and nitrogen dioxide. Our previous air pollution monitoring work in Chikhwawa has demonstrated that monitored CO exposure correlates weakly with COHb, PM_2.5_ exposure, and measured black carbon in airway cells from induced sputum.^20 45 53^

This study was conducted in the context of the largest cookstove intervention trial to date—a major strength enabling us to assess the effect of a cookstove intervention on childhood spirometry and air pollution exposure outcomes. Other strengths include high participation rates for spirometry and CO exposure monitoring, and good quality spirometry in a representative sample of children, despite the highly challenging research environment of a rural area in a low-income country. We achieved our sample size, even though field work was disrupted by vampirism hysteria in the community. We acknowledge limitations to our study including that personal monitoring of CO for 48 hours provides only a snapshot of exposure to a single pollutant. There are substantial limitations to the methods currently available for monitoring personal exposure to other pollutants in this young age group; the Lascar CO-monitoring device represents one of the best options available, at present. Monitoring during a 48 hours exposure period may not describe individual variation in daily and seasonal routines but reflected a compromise in terms of feasibility and acceptability in this large study population. Questionnaire data may have been subject to recall bias, with limited information on contributing factors such as birth weight, gestation at birth, HIV-status and exposure to passive smoking.

In conclusion, the substantial burden of chronic respiratory symptoms, abnormal spirometry and air pollution exposures in children in rural Malawi is concerning and calls for strategies to maximise healthy lung development and to effectively manage chronic respiratory conditions. To achieve this, research will be needed to develop ways to increase awareness of non-communicable lung diseases, such as asthma, at a community level to inform healthcare seeking behaviours and ensure access to appropriately trained healthcare providers and effective long-term treatment such as inhaled medication. Our finding of a potential beneficial effect of a cleaner burning biomass-fuelled cookstove on lung function (FVC) calls for further research into clean-air initiatives, tackling multiple sources of air pollution in a community-wide approach to promote lung health in children.

## References

[1] Asher I, Pearce N Global burden of asthma among children. int j tuberc lung dis 2014;18:1269–78. 10.5588/ijtld.14.0170 25299857

[2] Burney P, Jarvis D, Perez-Padilla R The global burden of chronic respiratory disease in adults. int j tuberc lung dis 2015;19:10–20. 10.5588/ijtld.14.0446 25519785

[3] GBD 2015 Chronic Respiratory Disease Collaborators Global, regional, and national deaths, prevalence, disability-adjusted life years, and years lived with disability for chronic obstructive pulmonary disease and asthma, 1990-2015: a systematic analysis for the global burden of Disease Study 2015. Lancet Respir Med 2017;5:691–706. 10.1016/S2213-2600(17)30293-X 28822787 PMC5573769

[4] Lai CKW, Beasley R, Crane J, et al Global variation in the prevalence and severity of asthma symptoms: phase three of the International study of asthma and allergies in childhood (Isaac). Thorax 2009;64:476–83. 19237391 10.1136/thx.2008.106609

[5] Ait-Khaled N, Odhiambo J, Pearce N, et al Prevalence of symptoms of asthma, rhinitis and eczema in 13- to 14-year-old children in Africa: the International study of asthma and allergies in childhood phase III. Allergy 2007;62:247–58. 10.1111/j.1398-9995.2007.01325.x 17298341

[6] Obaseki DO, Erhabor GE, Awopeju OF, et al Reduced forced vital capacity in an African population. Prevalence and risk factors. Ann Am Thorac Soc 2017;14:714–21. 10.1513/AnnalsATS.201608-598OC 28244800 PMC5427737

[7] Meghji J, Nadeau G, Davis KJ, et al Noncommunicable lung disease in sub-Saharan Africa. A community-based cross-sectional study of adults in urban Malawi. Am J Respir Crit Care Med 2016;194:67–76. 10.1164/rccm.201509-1807OC 26788760 PMC4960629

[8] Buist AS, McBurnie MA, Vollmer WM, et al International variation in the prevalence of COPD (the BOLD study): a population-based prevalence study. The Lancet 2007;370:741–50. 10.1016/S0140-6736(07)61377-4 17765523

[9] Burney P, Jithoo A, Kato B, et al Chronic obstructive pulmonary disease mortality and prevalence: the associations with smoking and poverty--a BOLD analysis. Thorax 2014;69:465–73. 10.1136/thoraxjnl-2013-204460 24353008 PMC3995258

[10] WHO Who guidelines for indoor air quality: household fuel combustion. Geneva, 2014. 25577935

[11] Mortimer K, Ndamala CB, Naunje AW, et al A cleaner burning biomass-fuelled cookstove intervention to prevent pneumonia in children under 5 years old in rural Malawi (the cooking and pneumonia study): a cluster randomised controlled trial. The Lancet 2017;389:167–75. 10.1016/S0140-6736(16)32507-7 PMC5783287 27939058

[12] Gordon SB, Bruce NG, Grigg J, et al Respiratory risks from household air pollution in low and middle income countries. The Lancet Respiratory Medicine 2014;2:823–60. 10.1016/S2213-2600(14)70168-7 25193349 PMC5068561

[13] FitzGerald JM, Carlsten C Respiratory disease associated with solid biomass fuel exposure in rural women and children: systematic review and meta-analysis. Thorax 2011;66:232–9. 10.1136/thx.2010.147884 21248322

[14] Wong GWK, Brunekreef B, Ellwood P, et al Cooking fuels and prevalence of asthma: a global analysis of phase three of the International study of asthma and allergies in childhood (Isaac). The Lancet Respiratory Medicine 2013;1:386–94. 10.1016/S2213-2600(13)70073-0 24429203

[15] Balmes JR, Eisen EA Household Air Pollution and Chronic Obstructive Pulmonary Disease. “A Riddle, Wrapped in a Mystery, Inside an Enigma”. Am J Respir Crit Care Med 2018;197:547–9. 10.1164/rccm.201801-0033ED 29373800

[16] Amaral AFS, Patel J, Kato BS, et al Airflow obstruction and use of solid fuels for Cooking or heating: bold results. Am J Respir Crit Care Med 2017. 10.1164/rccm.201701-0205OC PMC6005234 28895752

[17] Siddharthan T, Grigsby MR, Goodman D, et al Association between household air pollution exposure and chronic obstructive pulmonary disease outcomes in 13 low- and middle-income country settings. Am J Respir Crit Care Med 2018;197:611–20. 10.1164/rccm.201709-1861OC 29323928 PMC6005243

[18] Gauderman WJ, Vora H, McConnell R, et al Effect of exposure to traffic on lung development from 10 to 18 years of age: a cohort study. The Lancet 2007;369:571–7. 10.1016/S0140-6736(07)60037-3 17307103

[19] Postma DS, Bush A, van den Berge M Risk factors and early origins of chronic obstructive pulmonary disease. The Lancet 2015;385:899–909. 10.1016/S0140-6736(14)60446-3 25123778

[20] Nightingale R, Lesosky M, Flitz G, et al Non-communicable respiratory disease and air pollution exposure in Malawi (CAPS): a cross-sectional study. Am J Respir Crit Care Med 2018. 10.1164/rccm.201805-0936OC PMC6396863 30141966

[21] Rylance S, Nightingale R, Naunje A, et al Non-communicable lung disease and exposure to household air pollution in rural Malawian children: a cross-sectional study [abstract]. The International Journal of Tuberculosis and Lung Disease 2018;22.

[22] Kanyuka M, Ndawala J, Mleme T, et al Malawi and Millennium Development goal 4: a countdown to 2015 country case study. The Lancet Global Health 2016;4:e201–14. 10.1016/S2214-109X(15)00294-6 26805586

[23] Asher MI, Keil U, Anderson HR, et al International study of asthma and allergies in childhood (Isaac): rationale and methods. Eur Respir J 1995;8:483–91. 10.1183/09031936.95.08030483 7789502

[24] WHO Multicentre Growth Reference Study Group Who child growth standards: methods and development. Geneva, 2006.

[25] World Health organization and UNICEF Who child growth standards and the identification of severe acute malnutrition in infants and children. Geneva, Switzerland, 2009. 24809116

[26] Miller MR et al Standardisation of spirometry. European Respiratory Journal 2005;26:319–38. 10.1183/09031936.05.00034805 16055882

[27] Beydon N, Davis SD, Lombardi E, et al An official American thoracic Society/European Respiratory Society statement: pulmonary function testing in preschool children. Am J Respir Crit Care Med 2007;175:1304–45. 10.1164/rccm.200605-642ST 17545458

[28] Quanjer PH, Stanojevic S, Cole TJ, et al Multi-ethnic reference values for spirometry for the 3–95-yr age range: the global lung function 2012 equations. Eur Respir J 2012;40:1324–43. 10.1183/09031936.00080312 22743675 PMC3786581

[29] R Core Team R: A language and envronment for statistical computing [program]. 3.3.2 version. Vienna, Austria: R Foundation for Statistical Computing, 2013.

[30] WHO Who guidelines for indoor air quality: selected pollutants Bonn, 2010. 23741784

[31] Hooper LG, Dieye Y, Ndiaye A, et al Estimating pediatric asthma prevalence in rural Senegal: a cross-sectional survey. Pediatr Pulmonol 2016. 10.1002/ppul.23545 27551858

[32] Wa Somwe S, Jumbe-Marsden E, Mateyo K, et al Improving paediatric asthma care in Zambia. Bull. World Health Organ. 2015;93:732–6. 10.2471/BLT.14.144071 26600616 PMC4645426

[33] Kiboneka A, Levin M, Mosalakatane T, et al Prevalence of asthma among school children in Gaborone, Botswana. Afr H. Sci. 2016;16:809–16. 10.4314/ahs.v16i3.22 PMC5111997 27917215

[34] Mavale-Manuel S, Joaquim O, Macome C, et al Asthma and allergies in schoolchildren of Maputo. Allergy 2007;62:265–71. 10.1111/j.1398-9995.2006.01251.x 17298343

[35] Olaniyan T, Dalvie MA, Röösli M, et al Asthma-related outcomes associated with indoor air pollutants among schoolchildren from four informal settlements in two municipalities in the Western Cape Province of South Africa. Indoor Air 2019;29:89–100. 10.1111/ina.12511 30339304

[36] Oluwole O, Arinola GO, Huo D, et al Household biomass fuel use, asthma symptoms severity, and asthma underdiagnosis in rural schoolchildren in Nigeria: a cross-sectional observational study. BMC Pulm Med 2017;17 10.1186/s12890-016-0352-8 PMC5216579 28056916

[37] Lelijveld N, Seal A, Wells JC, et al Chronic disease outcomes after severe acute malnutrition in Malawian children (ChroSAM): a cohort study. The Lancet Global Health 2016;4:e654–62. 10.1016/S2214-109X(16)30133-4 27470174 PMC4985564

[38] Gray DM, Turkovic L, Willemse L, et al Lung function in African infants in the Drakenstein child health study. Impact of lower respiratory tract illness. Am J Respir Crit Care Med 2017;195:212–20. 10.1164/rccm.201601-0188OC 27509359 PMC5394784

[39] Hoo AF, Stocks J, Lum S, et al Development of lung function in early life: influence of birth weight in infants of nonsmokers. Am J Respir Crit Care Med 2004;170:527–33. 15172896 10.1164/rccm.200311-1552OC

[40] Latzin P, Roosli M, Huss A, et al Air pollution during pregnancy and lung function in newborns: a birth cohort study. European Respiratory Journal 2009;33:594–603. 10.1183/09031936.00084008 19010988

[41] Morales E, Garcia-Esteban R, Asensio de la Cruz O, et al Intrauterine and early postnatal exposure to outdoor air pollution and lung function at preschool age. Thorax 2015;70:64–73. 10.1136/thoraxjnl-2014-205413 25331281

[42] Gilliland FD et al Children's lung function and antioxidant vitamin, fruit, juice, and vegetable intake. American Journal of Epidemiology 2003;158:576–84. 10.1093/aje/kwg181 12965883

[43] Arigliani M, Canciani MC, Mottini G, et al Evaluation of the global lung initiative 2012 reference values for spirometry in African children. Am J Respir Crit Care Med 2017;195:229–36. 10.1164/rccm.201604-0693OC 27564235

[44] Rylance S, Mortimer K Galloping Hooves in Africa: horse, zebra. or Wildebeest? Ann Am Thorac Soc 2017;14:624–5. 28459627 10.1513/AnnalsATS.201701-061ED

[45] Havens D, Wang D, Grigg J, et al The cooking and pneumonia study (CAPS) in Malawi: a cross-sectional assessment of carbon monoxide exposure and carboxyhemoglobin levels in children under 5 years old. IJERPH 2018;15 10.3390/ijerph15091936 PMC6163876 30189674

[46] Romieu I, Riojas-Rodriguez H, Marron-Mares AT, et al Improved biomass stove intervention in rural Mexico: impact on the respiratory health of women. Am J Respir Crit Care Med 2009;180:649–56. 19556519 10.1164/rccm.200810-1556OC

[47] Smith-Sivertsen T, Díaz E, Pope D, et al Effect of reducing indoor air pollution on women's respiratory symptoms and lung function: the respire randomized trial, Guatemala. Am J Epidemiol 2009;170:211–20. 10.1093/aje/kwp100 19443665 PMC8889940

[48] Heinzerling AP, Guarnieri MJ, Mann JK, et al Lung function in woodsmoke-exposed Guatemalan children following a chimney stove intervention. Thorax 2016;71:421–8. 10.1136/thoraxjnl-2015-207783 26966237 PMC10666195

[49] Lee AG, Kaali S, Quinn A, et al Prenatal household air pollution is associated with impaired infant lung function with sex-specific effects: evidence from graphs, a cluster randomized Cookstove intervention trial. Am J Respir Crit Care Med 2018. 10.1164/rccm.201804-0694OC PMC6423100 30256656

[50] Oguonu T, Obumneme-Anyim IN, Eze JN, et al Prevalence and determinants of airflow limitation in urban and rural children exposed to cooking fuels in south-east Nigeria. Paediatr Int Child Health 2018;38:121–7. 10.1080/20469047.2018.1445506 29542392

[51] Herring MJ, Putney LF, Wyatt G, et al Growth of alveoli during postnatal development in humans based on stereological estimation. Am J Physiol Lung Cell Mol Physiol 2014;307:L338–L344. 10.1152/ajplung.00094.2014 24907055 PMC4137164

[52] Sturm R Theoretical models of carcinogenic particle deposition and clearance in children's lungs. J Thorac Dis 2012;4:368–76. 22934139 10.3978/j.issn.2072-1439.2012.08.03 PMC3426737

[53] Whitehouse AL, Miyashita L, Liu NM, et al Use of cleaner-burning biomass stoves and airway macrophage black carbon in Malawian women. Sci Total Environ 2018;635:405–11. 10.1016/j.scitotenv.2018.04.125 29677666 PMC6024563

