# Supplementary material for: Lung health and exposure to air pollution in Malawian children (CAPS): a cross-sectional study
Source: Thorax. 2019 Aug 29;74(11):1070–7. doi: 10.1136/thoraxjnl-2018-212945 (PMC6860406; doi:10.1136/thoraxjnl-2018-212945)
Supplement: Supplementary data [file thoraxjnl-2018-212945supp001.pdf]

Supplementary material

Table S1. Quality grading for spirometry

Quality grade	***FEV1 or FVC***
A	3 acceptable trials within 5% or 100ml
B	2 acceptable trials within 5% or 100ml
C	2 acceptable trials within 10% or 150ml
D	One acceptable trial
F	No acceptable trials

Table S2. Comparison of growth measurements and respiratory symptoms for children producing grade A-C and D or F spirometry traces

	A-C<br>N=522	D, F<br>N=280	p value *
Age, mean (SD) *years*	7.23 (0.78)	6.96 (0.72)	<0.001
**Growth parameters**			
Weight-for-age z-score, mean (SD)	-1.13 (0.89)	-1.04 (0.87)	0.17
Height-for-age z-score, mean (SD)	-1.04 (0.92)	-1.04 (0.87)	0.93
MUAC, mean (SD) *cm*	15.96 (1.28)	16.02 (1.23)	0.50
**Prevalence of chronic respiratory symptoms**			
Chronic cough, %	8.6	6.8	0.44
Current wheeze, %	7.3	6.8	0.91
Severe wheeze, %	4.2	3.2	0.61
Chronic SOB, %	6.7	5.0	0.42

*comparison of means using Student's t-test; comparison of proportions using Pearson's chi-squared test
